# Intradermal “Skin Boosters”: Are We Targeting Too Superficially?

**DOI:** 10.1111/jocd.70906

**Published:** 2026-05-18

**Authors:** Ting Song Lim, Suyeon Lee, Diala Haykal, Kyu‐Ho Yi

**Affiliations:** ^1^ Clique Clinic Kuala Lumpur Malaysia; ^2^ Medical Research Inc. Wonju Republic of Korea; ^3^ Centre Laser Palaiseau Palaiseau France; ^4^ You and I Clinic Seoul Republic of Korea

**Keywords:** bruising, erythema, hyaluronic acid, intradermal injection, pain, postinflammatory hyperpigmentation, skin booster, skin quality, subdermal injection

## 1 Introduction

“Skin booster” is an umbrella term applied to injectables intended to enhance skin quality rather than add contouring volume. These treatments are increasingly used in aesthetic practices to address concerns such as dullness, fine lines, and reduced skin elasticity without volumizing effects. Across regions it may refer to noncrosslinked or lightly crosslinked hyaluronic acid (HA) products, broader “biorevitalizers,” and other formulations, which complicates comparisons between products and study protocols [1]. Because “skin quality” spans visible attributes (skin tone and glow), mechanical properties (elasticity and firmness), and topographical features (texture and fine lines), standardized assessment frameworks have been recommended to improve interpretability and trial design [2, 3].

Intradermal microaliquot HA techniques are well established across multiple commercially available skin boosters. A randomized multicenter study of CPM‐HA20G (belotero Revive) showed significant improvements in skin hydration, elasticity, and roughness with favorable patient‐reported outcomes [4]. Similiarly, prospective study of VYC‐12 (Juvéderm Volite) demonstrated improvements in facial skin topography and hydration with expected local injection‐site responses such as erythema, edema, and bruising [5]. Systematic evidence syntheses also report meaningful improvements in hydration, elasticity, texture, and patient satisfaction with injectable HA, while emphasizing the need for larger randomized trials and consistent endpoints [6]. Across aesthetic practices, injection depth has also been recognized as a key technical variable influencing both efficacy and tolerability, with emerging consensus suggesting that different tissue planes may offer distinct biological and clinical advantages depending on treatment goals [7, 8]. In clinical practice, these techniques are typically delivered as multiple superficial microaliquots across the treatment area.

**FIGURE 1:**
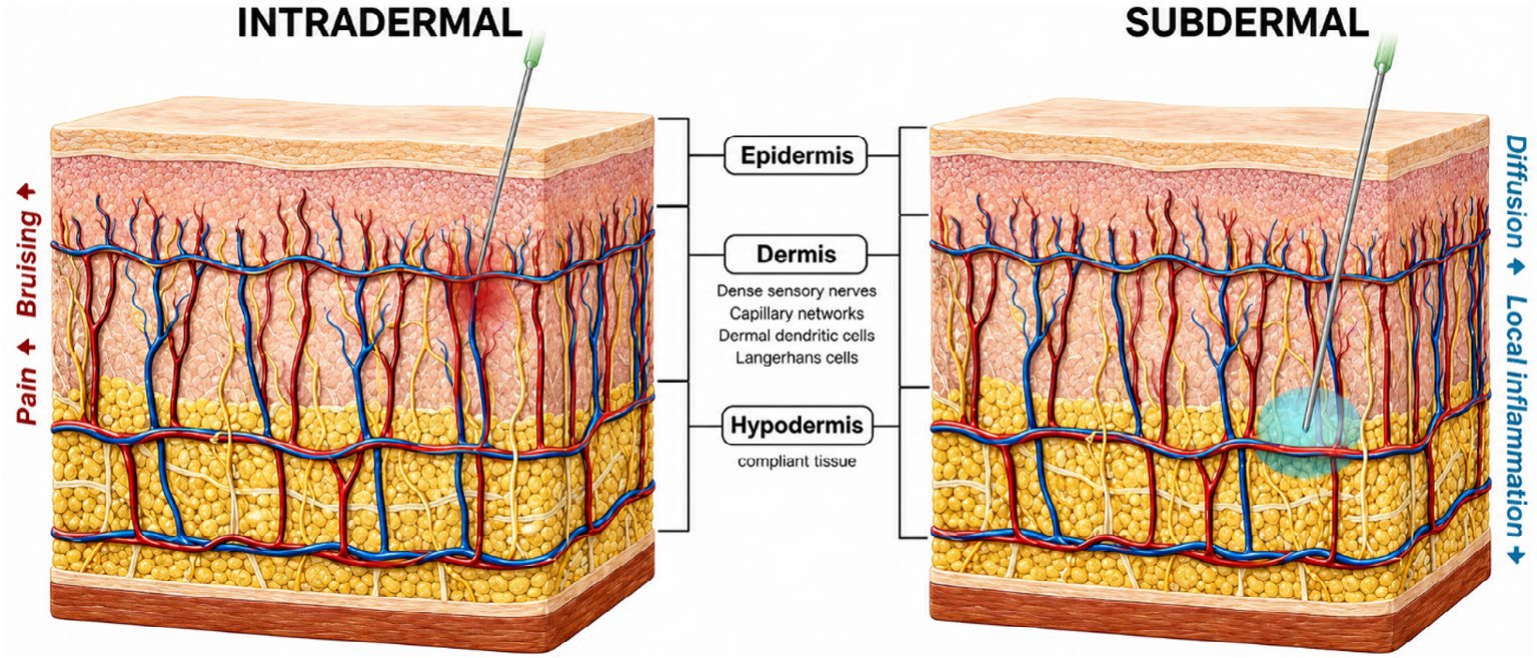
Comparison of intradermal and subdermal injection planes for skin‐quality treatments. Schematic illustration comparing intradermal and subdermal placement of injectable hyaluronic acid used for skin‐quality improvement. Intradermal injection deposits microaliquots within the superficial dermis, a tissue rich in sensory nerve endings, capillary networks, and immune cells such as dermal dendritic cells and Langerhans cells. Repeated superficial punctures may therefore be associated with increased pain, bruising, and visible inflammatory reactions. In contrast, subdermal injection places product immediately beneath the dermis at the dermohypodermal junction within the superficial subcutaneous tissue, which is more compliant and may permit broader product diffusion while reducing disruption of superficial nerves and capillaries.

The clinically relevant question is therefore not whether intradermal skin boosters can work, but whether the intradermal plane is always the most appropriate target for improving skin quality. If comparable skin‐quality outcomes can be achieved from a less reactive plane in selected patients and sites, depth selection could become a practical tool to reduce pain and downtime without sacrificing efficacy, highlighting the anatomical differences between intradermal and subdermal approaches (Figure 1).

### 1.1 Why Injection Depth Matters

#### 1.1.1 Innervation, Microvasculature, and Immune Activity

Injection depth shapes patient experience because the dermis and superficial subcutaneous tissue differ in innervation, vascular density, immune surveillance, and compliance.

##### 1.1.1.1 Innervation and Pain

Human skin is densely innervated, and superficial layers contain abundant sensory nerve endings involved in nociception and neuroimmune signaling [9]. These neural networks are closely integrated with local inflammatory pathways and can amplify pain perception during superficial tissue injury. In a blinded crossover trial, intracutaneous injections were significantly more painful than subcutaneous injections when the same volume was administered [10]. Clinically, intradermal microdroplet techniques often produce sharp stinging and transient wheals, especially when aliquots are injected quickly or under high pressure.

##### 1.1.1.2 Microvasculature and Bruising

The cutaneous microcirculation is organized into superficial and deep vascular plexuses connected by communicating vessels, with capillary loops supplying the papillary dermis [11]. Repeated intradermal punctures traverse capillary‐dense zones, increasing the likelihood of pinpoint bleeding, ecchymosis, and prolonged erythema. By contrast, subcutaneous tissue is generally more compliant and may permit broader distribution—particularly with cannulas—while reducing the number of dermal punctures.

##### 1.1.1.3 Immune Activity and Visible Inflammation

The dermis contains antigen‐presenting cells such as Langerhans cells and dermal dendritic cells, which can capture antigens introduced into skin [12]. In addition to sensory nerves and capillary networks, this immunologically active environment contributes to inflammatory signaling after tissue disruption. Although aesthetic injections are not immunizations, repeated intradermal microinjury occurs in an actively surveilled tissue and can predictably trigger erythema, edema, and papulation. This reactivity is usually transient, but it represents meaningful downtime for some patients and may contribute to pigmentary sequelae in predisposed individuals.

### 1.2 Technique Variables That Modify Intradermal Tolerability

Even when the intended target is intradermal, tolerability is strongly influenced by injection mechanics. A fluid‐mechanics analysis found that syringe and needle dimensions, needle length, and injectate viscosity affect the force required for intradermal delivery [13]. From a practical standpoint, lower injection pressure, smaller aliquots, slower delivery, and appropriate needle selection can reduce pain and whealing. However, if discomfort or visible reactivity remains a limiting factor despite optimization, shifting deposits toward the dermal–subcutaneous junction or into subcutaneous tissue may be a reasonable alternative in selected patients.

### 1.3 Is “Conditioning the Dermis From Below” Biologically Plausible?

**FIGURE 2:**
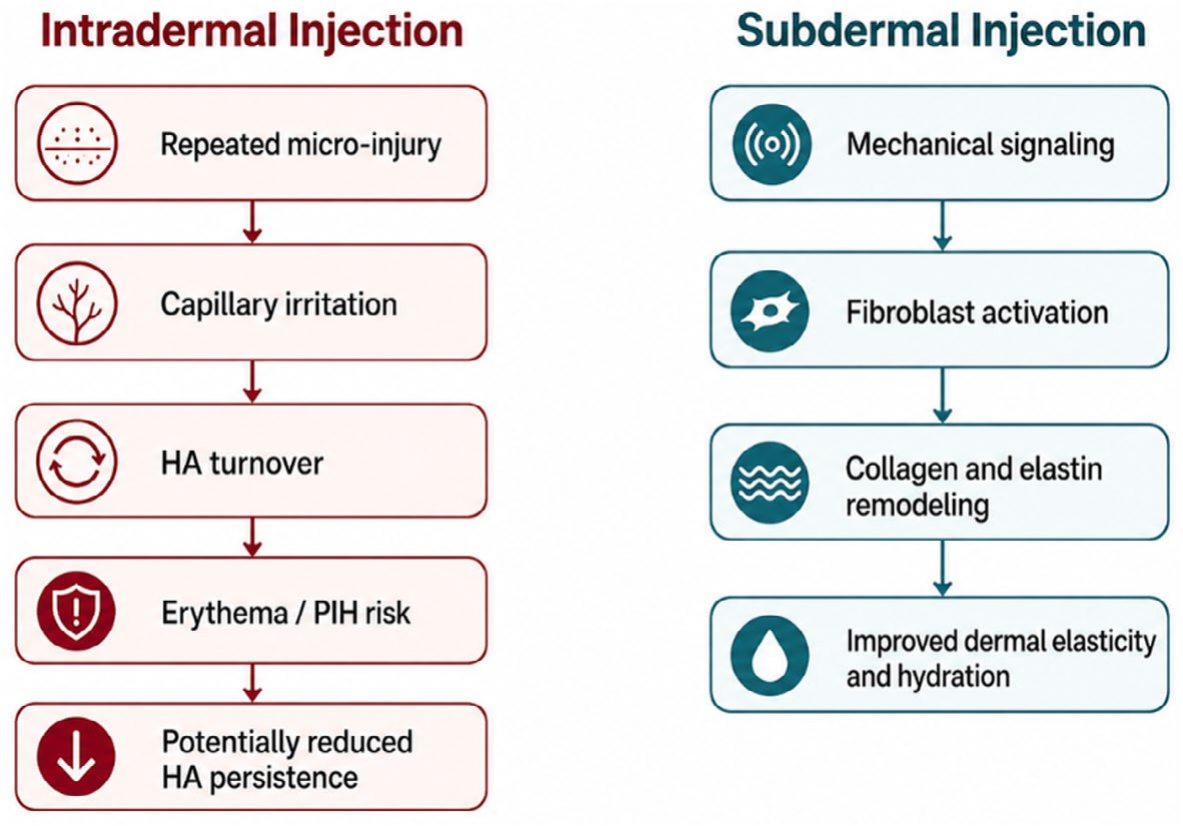
Conceptual pathways linking injection depth to biological responses and clinical outcomes. Proposed mechanisms by which injection depth may influence tissue responses and clinical outcomes in hyaluronic acid skin‐quality treatments. Intradermal injection involves repeated superficial microinjury and capillary irritation, which may promote local inflammatory signaling and increased hyaluronan turnover, potentially contributing to erythema, postinflammatory hyperpigmentation risk, and reduced persistence of injected HA. In contrast, subdermal placement may influence the overlying dermis through mechanical signaling at the dermal–subcutaneous interface, leading to fibroblast activation, extracellular matrix remodeling, and improvements in dermal elasticity and hydration. These mechanisms represent biologically plausible pathways based on current experimental and clinical observations.

The traditional rationale for intradermal HA is direct interaction with the dermal extracellular matrix to improve water binding and viscoelasticity. Yet dermal improvement may also be achievable from the adjacent subdermal plane. These concepts suggest that dermal improvement may occur through indirect biological pathways rather than direct dermal deposition alone (Figure 2).

First, the dermis and subcutaneous tissue function as a mechanically coupled unit. Mechanical interactions between the dermis and the underlying subcutaneous matrix may allow dermal fibroblasts to respond to strain and shear forces originating from adjacent tissues. These processes are mediated through mechanotransduction pathways, in which fibroblasts sense and convert mechanical stimuli into biochemical signals that regulate extracellular matrix production, collagen synthesis, and tissue remodeling. Such pathways have been increasingly recognized as key regulators of cutaneous homeostasis, inflammation, and repair. These observations support the biological plausibility that dermal remodeling could be influenced indirectly by injections delivered immediately beneath the dermis [14, 15].

Second, subcutaneous placement may reduce repeated injury to superficial nerves and capillaries, lowering bruising and persistent erythema while still influencing the overlying dermis through diffusion and interface signaling. Third, local inflammation may influence HA turnover. Human skin contains hyaluronidases and related proteins involved in HA degradation [16], and HA itself is increasingly recognized as an active regulator of inflammatory signaling in tissue injury and repair [17]. Beyond its structural role, HA fragments of different molecular weights can exert distinct biological effects, including modulation of cytokine release and immune cell activation. These interactions suggest that repeated superficial inflammatory stimulation may influence both the degradation kinetics and biological activity of injected HA. These observations do not establish that intradermal placement shortens longevity, but they support a testable hypothesis: techniques that minimize superficial inflammatory activation may improve the patient experience and possibly influence duration of effect for certain products.

### 1.4 Clinical Evidence for Subdermal Skin‐Quality Improvement

Evidence for subdermal HA approaches is less mature than for intradermal delivery but is steadily emerging. In a clinical and ultrasound‐based evaluation of two non‐crosslinked HA products delivered via cannula, patients reported minimal pain with only mild, transient bruising or edema; ultrasound findings demonstrated dermal thickening and hydration‐related changes as early as 3 weeks post‐treatment [18]. Although limited by short follow‐up, these findings support the feasibility of achieving measurable skin‐quality improvements with reduced surface trauma.

Real‐world clinical reports further suggest that practitioners may preferentially use cannula‐based delivery to place skin boosters within subcutaneous layer, aiming to enhance skin quality while minimizing bruising and contour irregularities in anatomically sensitive regions [19]. This reflects a broader shift in aesthetic practice toward technique individualization, where injection depth and delivery method are adopted to optimize efficacy, patient comfort and downtime [8].

Importantly, converging evidence from adjacent regenerative approaches reinforces the biological plausibility of subcutaneous‐driven dermal remodeling. A prospective clinical study of Polynucleotides PN‐HPT in Asian patients demonstrated significant improvements in skin elasticity, hydration, and overall quality following cannula delivery, supporting the concept that extracellular matrix modulation and fibroblast activation can be initiated from deeper tissue planes [4]. Similarly, in a prospective trial of hyperdiluted calcium hydroxylapatite for neck rejuvenation, clinical improvement correlated with ultrasound‐documented increases in dermal thickness over time [20].

Collectively, these findings support a pragmatic and evolving paradigm: the dermis can be effectively influenced from below, and injection depth may be strategically selected to balance clinical efficacy with tolerability, safety and downtime.

### 1.5 Practical Clinical Decision‐Making

Depth selection should be individualized rather than treated as a single “correct” plane. In practice, intradermal delivery may be preferred when direct, diffuse dermal hydration and fine textural improvement are the primary treatment goals, paticularly in patients who are willing to accept mild, transient visible reactivity. In contrast, a subcutaneous (subdermal) approach may be advantageous in patients with lower pain tolerance, higher risk of bruising, or a propensity toward post‐inflammatory hyperpigmentation (PIH). This consideration is especially relevant in individuals with more reactive or inflammtion‐prone skin phenotypes, such as many Asian skin types, where repeated dermal puncture may case prolonged erythema and pigmentary sequelae.

Subdermal delivery may also be preferred in clinical scenarios where minimizing downtime is a priority, or in anatomically sensitive areas (e.g., neck, periorbital region) where repeated dermal puncture is less desirable. These considerations support a patient‐centered approach in which injection depth is selected based on both biological rationale and individual patient priorities [21, 22]. Anatomic site matters: papules and bruising are often more conspicuous on the neck and in thin‐skinned regions, whereas the cheeks may tolerate intradermal microaliquots more easily. Product and device choices also matter—lower‐viscosity products, smaller aliquots, and slow low‐pressure delivery generally improve distribution; cannulas can reduce puncture burden but require consistent plane control.

### 1.6 Safety Considerations

Comfort and downtime must be clearly distinguished from safety. While subdermal placement may reduce superficial reactivity, it does not inherently confer a lower risk profile. Subdermal facial planes can contain larger‐caliber vessels, and inadvertent intravascular injection of HA carries a risk of vascular occlusion and serious complications. Therefore, deeper placement is not automatically safer; rather, it introduces a different set of anatomical considerations and technical challenges. Regardless of injection depth, clinicians must prioritize detailed knowledge of regional vascular anatomy, careful product selection and dosing, slow low‐pressure injection techniques, and continuous reassessment during delivery.

### 1.7 Research Priorities

Randomized split‐face studies using the same HA product, dose, and standardized technique are well suited to determine whether subdermal delivery can match intradermal outcomes while improving tolerability. Key endpoints should include pain scores, objective bruising/erythema, downtime duration, and standardized skin‐quality measures consistent with published frameworks [2]. Patient‐reported outcomes (comfort, satisfaction, social downtime) should be captured alongside objective measurements.

Pigmentary outcomes should be explicitly assessed in higher‐risk phototypes, including standardized photography and PIH grading at 4–12 weeks [21, 22]. High‐frequency ultrasound can add objective measures of deposition plane, dermal thickness, and structural change, which are particularly relevant for depth‐comparison studies [18, 20]. Longer follow‐up is necessary to test durability hypotheses and to assess whether depth influences the interval to retreatment.

## 2 Conclusion

Intradermal HA skin boosters are effective for improving skin quality, but the dermis' dense innervation, microvasculature, and immune activity make superficial delivery predictably painful and reactive for many patients. Emerging clinical and mechanistic evidence suggests that superficial subcutaneous HA placement may provide comparable skin‐quality benefits in selected patients while reducing discomfort and visible inflammation. Rather than representing competing approaches, intradermal and subdermal techniques may be viewed as complementary tools within a depth‐based treatment strategy. Future comparative studies are needed to better define patient selection, anatomical considerations, and product‐specific outcomes across different injection planes [23, 24, 25, 26, 27, 28].

While intradermal approaches remain the traditional standard for skin booster delivery, subdermal techniques may offer a complementary strategy in selected clinical scenarios where reduced reactivity and improved tolerability are prioritized.

## Author Contributions

All authors have reviewed and approved the article for submission. Conceptualization: Ting Song Lim, Suyeon Lee, Diala Haykal, Kyu‐Ho Yi. Writing – original draft preparation: Ting Song Lim, Diala Haykal, Kyu‐Ho Yi. Writing – review and editing: Ting Song Lim, Suyeon Lee, Diala Haykal, Kyu‐Ho Yi. Visualization: Ting Song Lim, Suyeon Lee, Diala Haykal, Kyu‐Ho Yi. Supervision: Kyu‐Ho Yi.

## Funding

The authors have nothing to report.

## Ethics Statement

The authors have nothing to report.

## Consent

The authors have nothing to report.

## Conflicts of Interest

The authors declare no conflicts of interest.

## Data Availability

Data sharing not applicable to this article as no datasets were generated or analysed during the current study.
